# Supplementary material for: DMRforPairs: identifying Differentially Methylated Regions between unique samples using array based methylation profiles
Source: BMC Bioinformatics. 2014 May 15;15:141. doi: 10.1186/1471-2105-15-141 (PMC4046028; doi:10.1186/1471-2105-15-141)
Supplement: Additional file 2 — DMRforPairs output for the comparison of A431-MCF7 and NA17018-NA17105. Please start from the HTML files in each folder. Available via the BMC Bioinformatics website. [file 1471-2105-15-141-S2.zip › 1394847754114233_MOESM2_ESM/A431_MCF7/figures/12170.pdf]

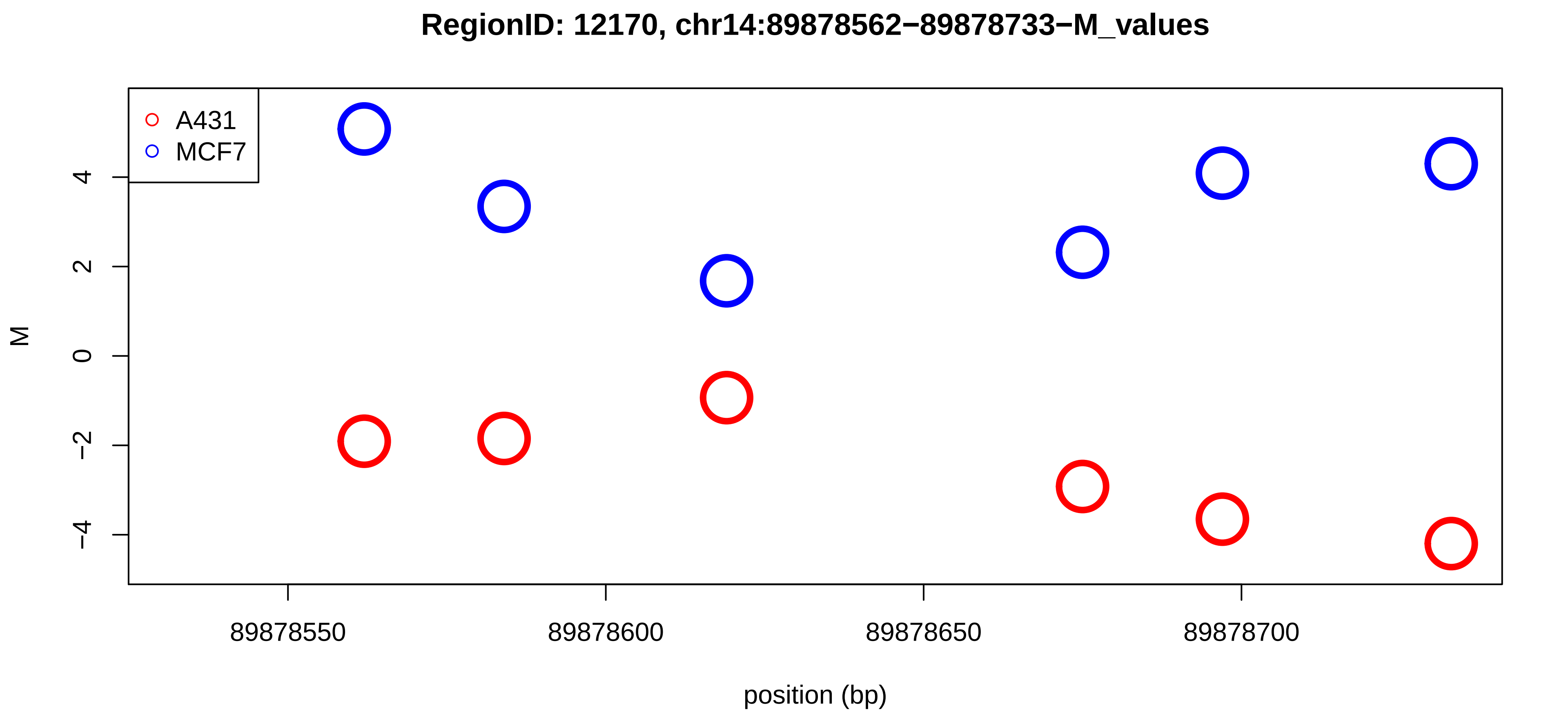
RegionID: 12170, chr14:89878562−89878733−M_values
A431
MCF7
M
4
2
0
−2
−4
89878550
89878600
89878650
89878700
position (bp)

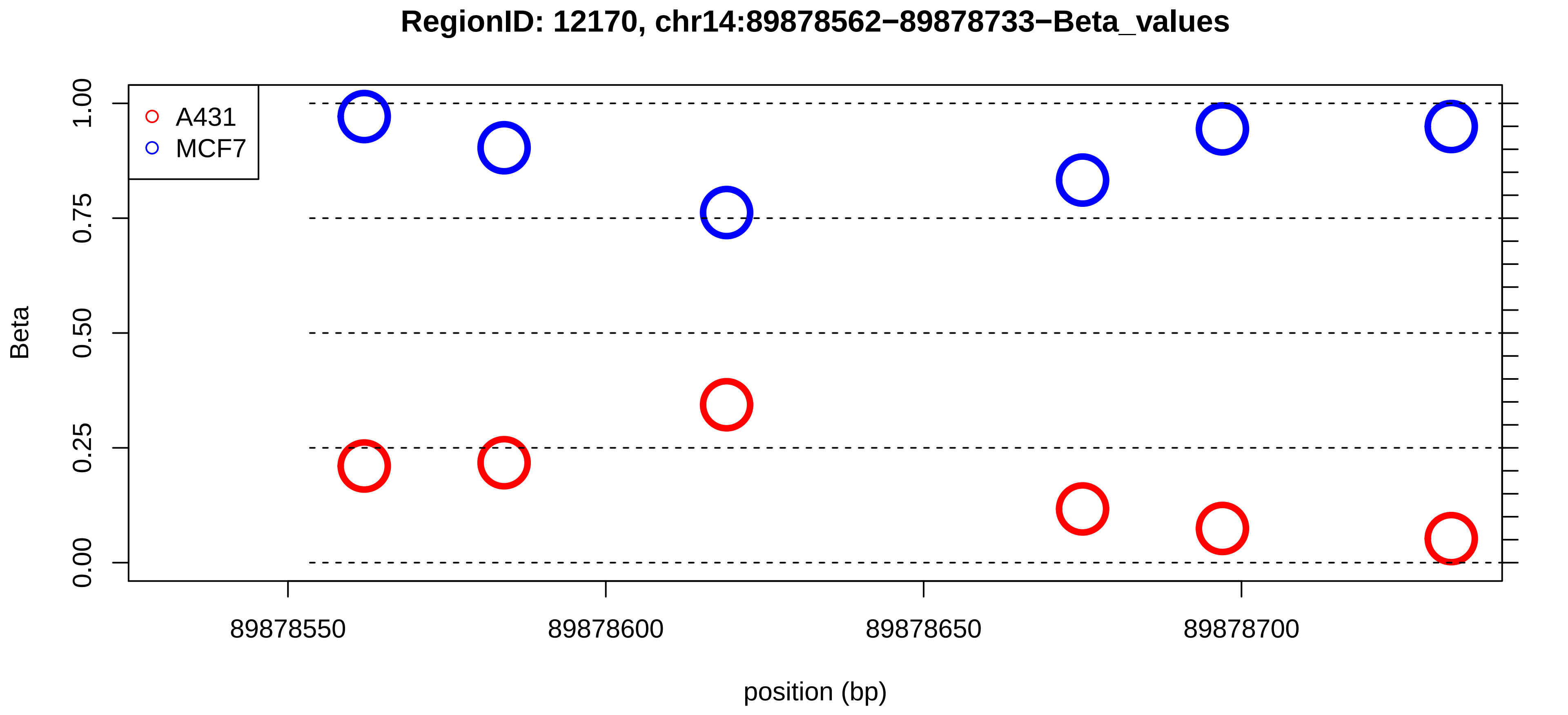
RegionID: 12170, chr14:89878562−89878733−Beta_values
A431
MCF7
Beta
1.00
0.75
0.50
0.25
0.00
89878550
89878600
89878650
89878700
position (bp)

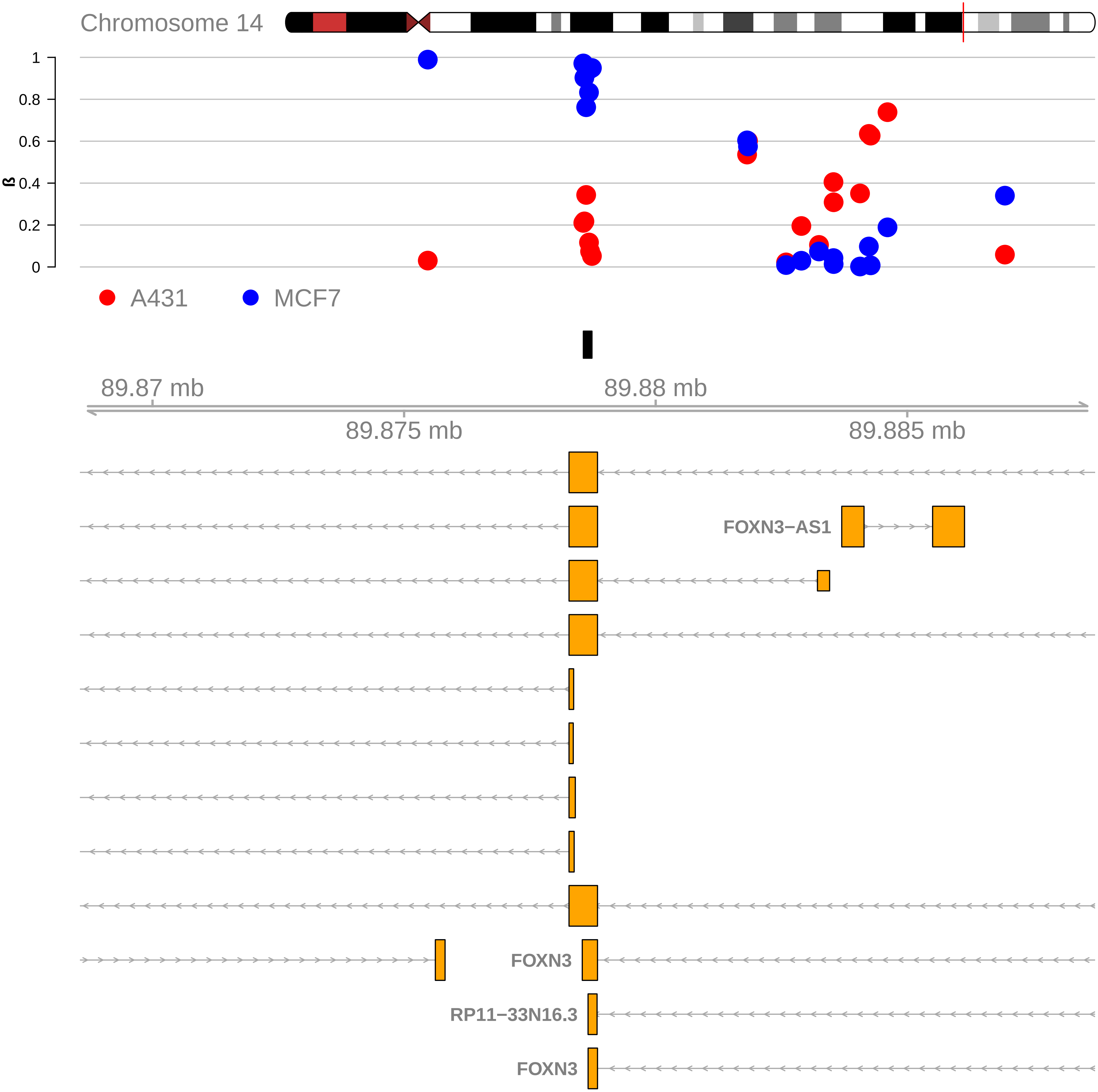

Chromosome 14
1
0.8
0.6
ß 0.4
0.2
0
A431
MCF7
89.87 mb
89.88 mb
89.875 mb
89.885 mb
FOXN3−AS1
FOXN3
RP11−33N16.3
FOXN3
